# Sox10 Expression in Goldfish Retina and Optic Nerve Head in Controls and after the Application of Two Different Lesion Paradigms

**DOI:** 10.1371/journal.pone.0154703

**Published:** 2016-05-05

**Authors:** Marta Parrilla, Fernando León-Lobera, Concepción Lillo, Rosario Arévalo, José Aijón, Juan Manuel Lara, Almudena Velasco

**Affiliations:** 1 Instituto de Neurociencias de Castilla y León, Universidad de Salamanca, Salamanca, Spain; 2 IBSAL, Salamanca, Spain; Instituto Murciano de Investigación Biosanitaria-Virgen de la Arrixaca, SPAIN

## Abstract

The mammalian central nervous system (CNS) is unable to regenerate. In contrast, the CNS of fish, including the visual system, is able to regenerate after damage. Moreover, the fish visual system grows continuously throughout the life of the animal, and it is therefore an excellent model to analyze processes of myelination and re-myelination after an injury. Here we analyze Sox10^+^ oligodendrocytes in the goldfish retina and optic nerve in controls and after two kinds of injuries: cryolesion of the peripheral growing zone and crushing of the optic nerve. We also analyze changes in a major component of myelin, myelin basic protein (MBP), as a marker for myelinated axons. Our results show that Sox10^+^ oligodendrocytes are located in the retinal nerve fiber layer and along the whole length of the optic nerve. MBP was found to occupy a similar location, although its loose appearance in the retina differed from the highly organized MBP^+^ axon bundles in the optic nerve. After optic nerve crushing, the number of Sox10^+^ cells decreased in the crushed area and in the optic nerve head. Consistent with this, myelination was highly reduced in both areas. In contrast, after cryolesion we did not find changes in the Sox10^+^ population, although we did detect some MBP^-^ degenerating areas. We show that these modifications in Sox10^+^ oligodendrocytes are consistent with their role in oligodendrocyte identity, maintenance and survival, and we propose the optic nerve head as an excellent area for research aimed at better understanding of de- and remyelination processes.

## Introduction

The transcription factor Sox10 belongs to the Sox family and is characterized by the high-mobility DNA-binding HMG domain. Sox10, together with Sox8 and Sox9, form the SoxE subfamily. During the development of the central nervous system (CNS) SoxE proteins promote the formation, differentiation and survival of the oligodendrocyte lineage, from OPCs to myelinating oligodendrocytes [1,2]. Studies performed in *Sox10* mutant mice and mutant zebrafish have shown that *Sox10* is necessary for oligodendrocyte differentiation during development [3,4]. Moreover, highly conserved enhancer elements of *SOX10* have allowed the identification of human oligodendrocyte precursor cells (OPCs) and oligodendrocyte lineage cells in cell cultures [5]. Sox10 also promotes the expression of some myelin genes [1,2], such as myelin basic protein (MBP), the second most abundant protein in the CNS [6], which plays a fundamental role in myelin formation [7,8]. Sox10 is thought to promote the gene expression of factors that mediate oligodendrocyte-axon interactions, which are necessary for oligodendrocyte survival during development [4]. Finally, Sox10 is also involved in the myelination of the peripheral nerve system (PNS) during development [2]. In the adult CNS, Sox10 continues to be expressed by mature oligodendrocytes, maintaining their oligodendroglial phenotype [2].

During the development of the vertebrate visual system, OPCs originating in the preoptic area migrate along the optic nerve (ON) towards the retina [9,10]. In many species of mammals, OPCs stop migrating when they reach the *lamina cribosa*, located in the optic nerve head (ONH) [11–13]. However, in fish [14–19], reptiles [16,20,21], birds [22–24] and some mammals, such as rabbits [19,25–27], oligodendrocytes cross the ONH, reach the retina, and myelinate retinal ganglion cell (RGC) axons. Nonetheless, this myelination occurs in a peculiar way, since the myelin sheaths exhibit a loose appearance [17,21,23,26,28].

The visual system of adult fish grows continuously due to the addition of retinal cells from the periphery of the retina, called the peripheral growth zone (PGZ). Moreover, the fish visual system can regenerate after suffering a lesion [29,30], in contrast to mammals, whose axons have a very low capacity for regeneration [31]. Thus, nascent axons from the new RGCs can be detected constantly, growing from the PGZ towards the ONH, where they converge to form the ON. When the PGZ is destroyed [32,33], Pax2 astrocytes in the ONH react and proliferate after the arrival of newly born axons from the PGZ [33]. In addition, when the ON is crushed and severed RGC axons are re-growing, the astrocytes and oligodendrocytes of the ONH are respectively responsible for their guidance and packaging [34] and their myelination once they have reached the optic tectum [35,36].

Despite the large body of information available regarding fish ONH astrocytes, little is known about retinal and ONH oligodendrocytes. Thus, the present work aims to clarify the identity of these cells. Moreover, we analyze the response of oligodendrocytes in the absence of newly formed RGCs when the PGZ is destroyed and the new axonal input is lost, comparing this with a well-established model for axonal degeneration, ON crushing.

## Material and Methods

### Animals

We used adult 8-12-cm long goldfish (*Carassius auratus*) obtained from commercial suppliers. All animals were kept in aquaria at 18±1°C on a 12 h light/dark cycle; they were daily fed and, prior to analysis, were deeply anesthetized with 0.03% tricaine methanesulfonate in water (MS-222; Sigma). All experimental procedures were in accordance with the guidelines of the European Union Council Directive (2010/63/EU) and current Spanish legislation for the use and care of animals (RD 53/2013). Also, all procedures involving animals were assessed and approved by the Animal Ethical Committee of the University of Salamanca before commencing the experimentation in the centres associated with the Experimentation Service.

### Lesion paradigms

Both cryolesion of the retinal PGZ and ON crushing were carried out in adult goldfish as previously described [37,38]. In brief, the cryolesion of the PGZ was performed using a cryo-application system that employs liquid nitrogen to freeze a metallic probe. A 1 mm-wide probe was used to induce the lesion in the peripheral part of the retina of the right eye. In the ON crush experiments, the lesion was performed in the left ON, 1 mm away from the eyeball with a fine watchmaker`s forceps for about 3 seconds.

A total number of 100 fish were sacrificed at 2, 7, 15, 21, 30, 60, 90, 120, 180 and 210 days post-injury (d).

### Immunohistochemistry

Four animals from each time-point analyzed were perfused transcardially with a NaCl solution followed by 4% paraformaldehyde (PFA) and 0.2% picric acid in 0.1 M phosphate buffer, pH 7.4 (PB). Their eyes were dissected out and post-fixed for 2 h at room temperature (RT) in the same fixative solution, then washed in PB, cryoprotected in 50% sucrose, and 14-μm transverse sections were obtained in a cryostat.

After washes with 0.1 M phosphate buffered saline (PBS), pH 7.4, with 0.02% Triton X-100 (PBS-Tx), sections were post-fixed with 100% methanol for 5 min and auto-fluorescence was quenched with 2.5 g/L NaBH_4_ in PBS. Subsequently, sections were blocked with 2% normal goat serum (2h) in PBS-Tx and primary antibodies (Sox10, MBP, Pax2, PCNA and Zn8) were incubated overnight in 1% DMSO PBS-Tx-serum (Table 1). After washing in PBS, sections were incubated 1h at RT with 1:250 Cy2 and Cy3 fluorescent secondary antibodies (Jackson). PCNA labeling was enhanced using a secondary biotinylated antibody (Vector, 1:250) followed by streptavidin Cy2 (Jackson) (1:200). Nuclei were counterstained with DAPI (Sigma) diluted 1:10.000. Negative controls without primary or secondary antibodies were performed. Sections were mounted with an anti-fading mixture (0.42 g glycine, 0.021 g NaOH, 0.51 g NaCl, 1 mL thimerosal 2%, 5 g N-propyl gallate; 300 mL distilled water and 70 mL glycerine).

**Table 1:** Antibodies used in the present work.

Antibody	Dilution	Supplier	Host	Catalogue number/Reference
Pax2	1:900	Covance	Rabbit polyclonal	PRB-276P
Sox10	1:1000	Dr. Bruce Appel	Rabbit polyclonal	Takada et al, 2010
MBP	1:1000	Dr. Bruce Appel	Rabbit polyclonal	Takada et al, 2010
PCNA	1:500	Santa Cruz Biotech	Mouse monoclonal	sc-56
Zn8	1:300	Dev. Studies Hybridoma Bank	Mouse monoclonal	Zn-8

For whole-mount retinal staining, fish were dark-adapted for 2 h and then sacrificed. Retinas were dissected out maintaining their orientation from 3 animals per group, fixed for 4h in 4% PFA, and thoroughly washed in PBS. Immunostaining was performed as described above, except that the primary antibodies were incubated for 4 days at 4°C and secondary antibodies were incubated overnight at 4°C.

### Image analysis

ONH cell quantification was performed as previously described [33,34]. We counted cells from 2–6 tissue sections randomly selected from 4 control and 4 injured animals from each survival time (2-210d). We counted all labeled cells present in the ONH, which is the area located between the two edges of the neural retina, excluding the central artery [39]. In whole-mount retinas, cells were counted from 3 retinas from both control and injured animals. All cell counts were performed manually with ImageJ software. Statistical analysis were carried out with the Graph Prism software. ANOVA test was run after checking data normality and homoscedasticity. Dunnet’s post hoc test was performed to show the differences between control and injured groups.

### Electron microscopy

Two animals from each time-point analyzed were perfused transcardially with NaCl solution followed by 4% PFA, 0.2% picric acid and 0.25% glutaraldehyde in PB. The ONH was dissected out and post-fixed with 4% PFA and 0.25% glutaraldehyde in PB for 2 h at RT. After several washes in PB, the samples were fixed with 0.5% aqueous osmium tetroxide, dehydrated and flat-embedded in Epon 12 resin, as described previously [33,39]. Ultrathin sections were stained with 2% aqueous uranyl acetate and lead citrate.

### Photomicrographs

Some of the light microscopy and fluorescence images were obtained with an Olympus AX-70 microscope and the other fluorescence images were obtained with a laser scanning spectral confocal microscope (Leica TCS SP2). Ultrathin sections were observed under a Zeiss EM900 electron microscope and pictures were taken with a digital camera connected to the microscope using ImageSP software. The brightness and contrast of the original images were further processed with Adobe Photoshop CS4 software.

## Results

### Location and distribution of Sox10^+^ cells in the goldfish retina and ONH

**Fig 1:**
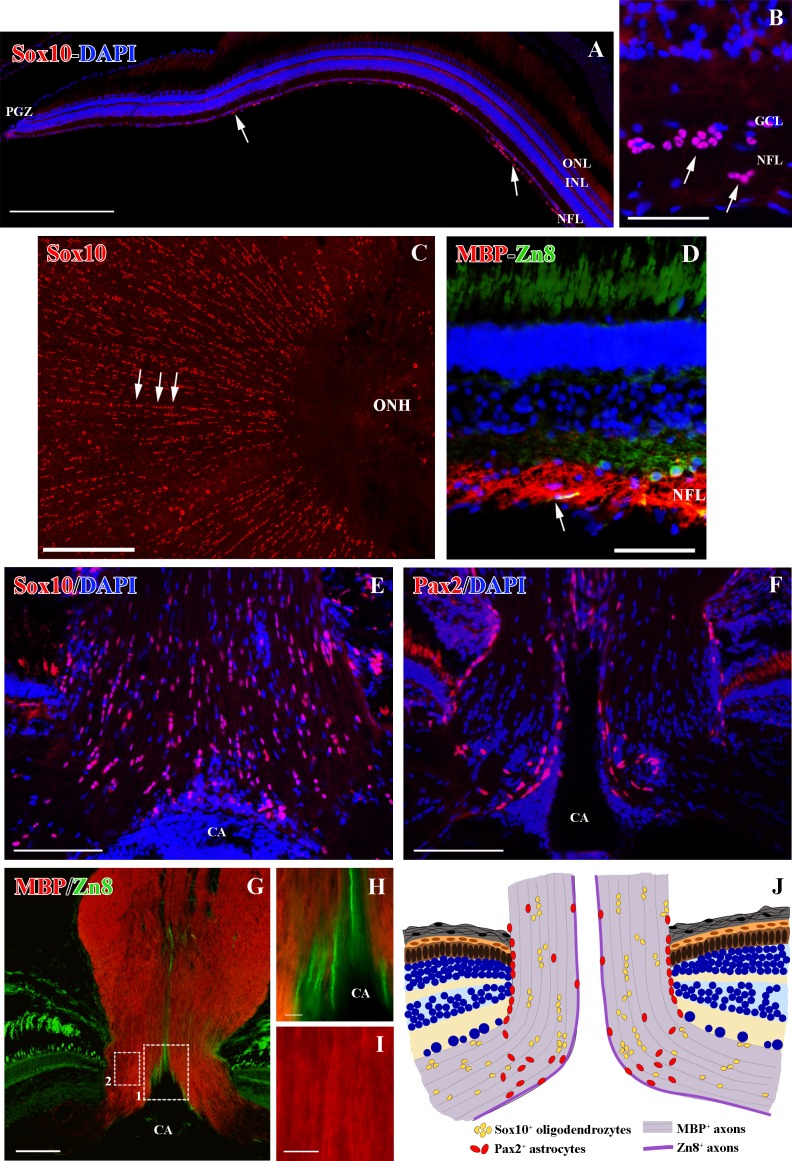
Distribution of Sox10^+^ cells in the goldfish retina and ONH. **(A)** Sox10^+^ cells distribution from the peripheral to the central retina (arrows). **(B)** Sox10^+^ cells in the retina close to the ONH (arrows). **(C)** Whole-mount retina showing Sox10^+^ cells arranged in groups and rows (arrows). **(D)** Cross section of the retina close to the ONH showing MBP^+^ axons and growing Zn8^+^ axons (arrow). **(E)** Sox10^+^ cells in the ONH. **(F)** Pax2^+^ cells in the ONH. **(G-I)** ONH MBP^+^ axons and growing Zn8^+^ axons. Squares 1 and 2 enlarged in H and I respectively. **(J)** ONH scheme, comparing the distribution of both Sox10^+^ cells and Pax2^+^ astrocytes. CA: central artery, GCL: ganglion cell layer, INL: inner nuclear layer, NFL: nerve fiber layer, ONH: optic nerve head, ONL: outer nuclear layer. Scale bars: A, C: 200 μm;, E-G: 100 μm; B, D: 50 μm; H-I: 10 μm.

We found that Sox10 immunolabeling was located exclusively in the nuclei of the cells, which were distributed from the retina (Fig 1A–1C) along the whole length of the ON (Fig 1E, see section 2.5).

In the retina, Sox10^+^ cells were located in the nerve fiber layer (NFL) from regions close to the PGZ (Fig 1A) to the central retina (Fig 1B and 1C). However, the number of Sox10^+^ cells in the peripheral retina was much lower than in the central retina (Fig 1A and 1B). In the central retina they were located close to the RGCs in the ganglion cell layer (GCL) and also in vitreal parts of the NFL (Fig 1B). Similar results were found for the myelin protein marker MBP. We detected a low amount of MBP^+^ labeling in the PGZ, where young, newly formed Zn8^+^ RGC axons were located (S1 Fig). However, in the central retina MBP^+^ labeling was higher and was distributed throughout the whole thickness of the NFL, whereas the Zn8^+^ RGC axons remained in the vitreal part of this layer (Fig 1D).

In the ONH, Sox10^+^cells were numerous and were organized in groups and in rows (Fig 1C and 1E). They were distributed throughout the whole ONH, but they did not form part of the glia limitans. This organization and distribution differed from Pax2^+^ astrocytes, which were mostly located in the glia limitans of the central artery (CA) and in the retina-ONH transition (Fig 1F). By contrast, MBP was located throughout the ONH, with a much more intense staining and organization than in the retina (Fig 1G and S1 Fig). While Zn8^+^/MBP^-^ RGC axons were located close to the CA in the growing edge (Fig 1H), well-organized and compacted MBP^+^ myelin sheaths were located in the mature ONH (Fig 1I). Fig 1J summarizes the distribution of both Pax2^+^ astrocytes and Sox10^+^ cells in the goldfish ONH.

**Fig 2:**
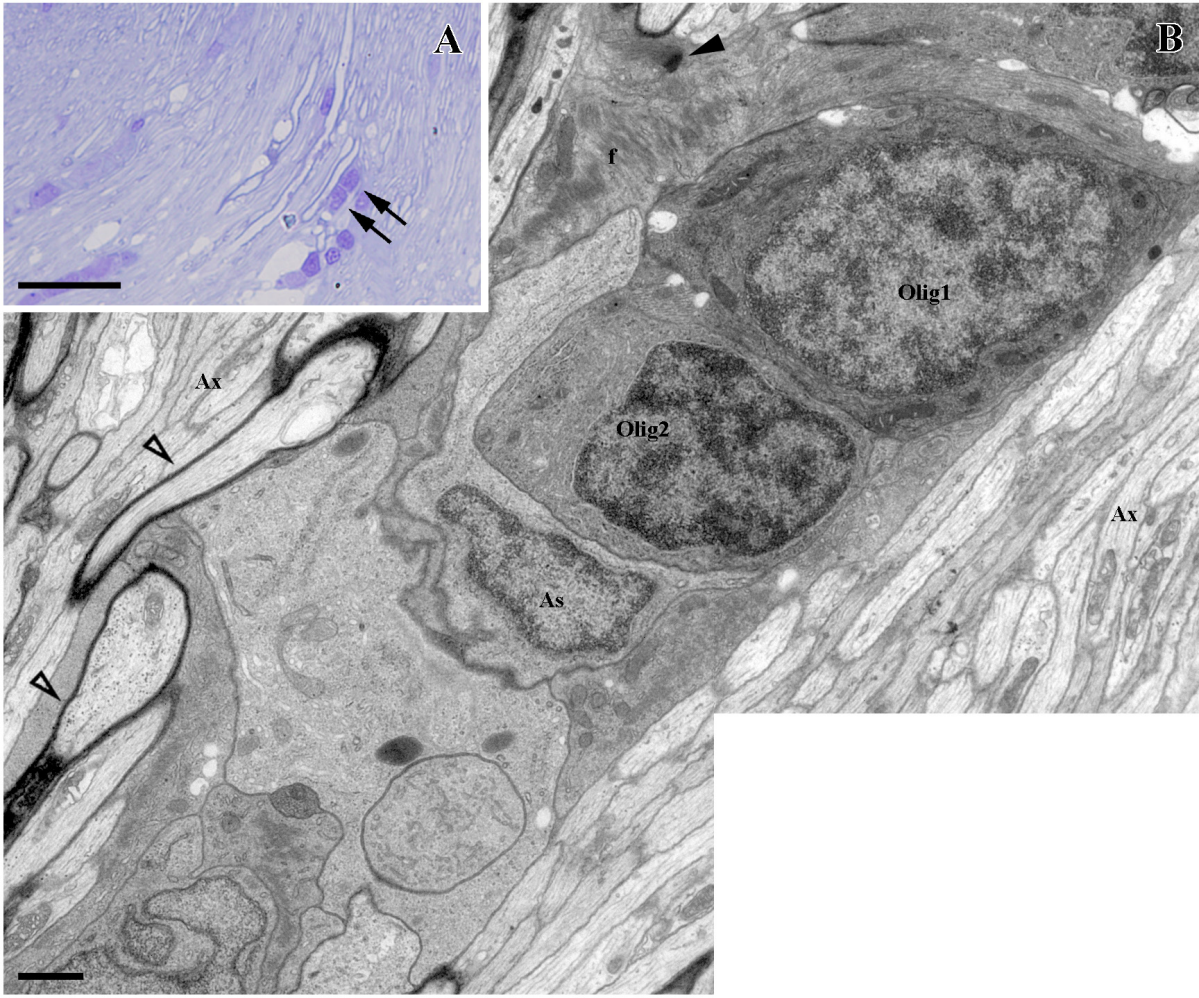
Ultrastructural study of control ONH. **(A)** Cells arranged in rows (arrows). **(B)** Two oligodendrocytes arranged in rows (Olig1 and Olig2) enveloped by astrocytes (As) and containing intermediate filaments in their cytoplasm (f) and joined by desmosomes (black arrowhead). Myelinated (empty arrowheads) and unmyelinated axons (Ax) are indicated in the image. Scale bars: A: 10 μm, B: 1000 nm.

In semithin sections we found these oligodendrocytes arranged in rows, and we analyzed their ultrastructure by electron microscopy (Fig 2A and 2B). They showed a heterochromatic round nucleus and scarce cytoplasm, both classical characteristics of oligodendrocytes. They never showed the typical features of astrocytes, i.e., intermediate filaments or desmosomes (Fig 2B). Moreover, the oligodendrocytes were enveloped by astrocyte intermediate filament-filled processes, and were located close to myelinated and unmyelinated axons (Fig 2B).

### Sox10^+^ ONH oligodendrocytes after PGZ cryolesion

**Fig 3:**
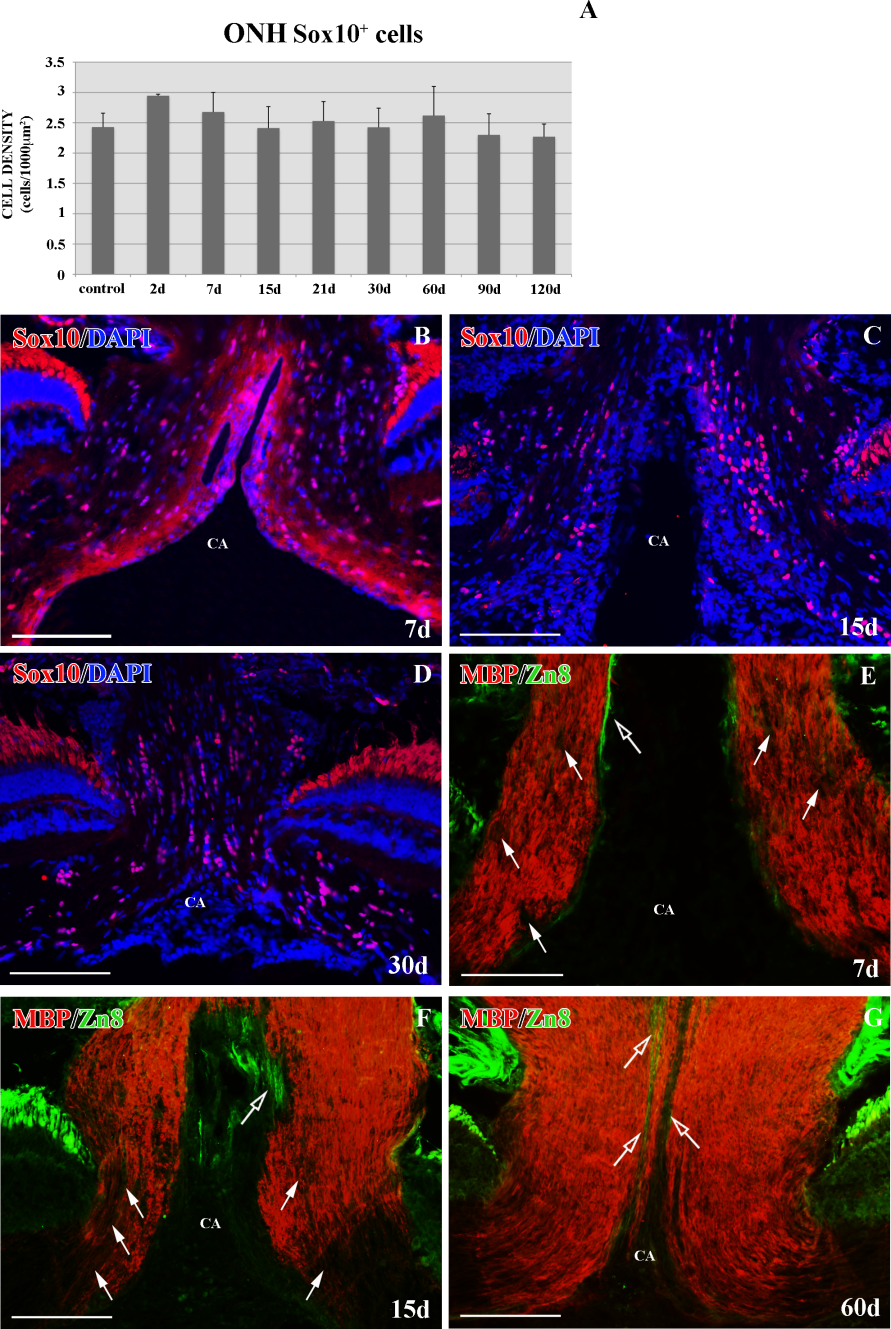
Sox10^+^ cells and MBP^+^ axons in the ONH after PGZ cryolesion. Cells were counterstained with DAPI. **(A)** Statistical analysis of Sox10^+^ cell density in the ONH after PGZ cryolesion. No significant differences were found. Data are shown as means± standard deviations. n = 4 animals per group. **(B-D)** Sox10^+^ cells from 7d, 15d and 30d after injury. **(E-G)** MBP^+^ axons with degenerating areas (arrows) and growing Zn8^+^ axons (empty arrows) from 7d, 15d and 60d. CA: central artery. Scale bars: B-G: 100 μm.

Previous studies carried out at our laboratory revealed the relevance of the ONH Pax2^+^ astrocytes when the PGZ was eliminated. In the present study, we wished to analyze the effect of the loss of this axonal input on Sox10^+^ ONH cells. To investigate their behavior, we removed this area by cryolesion. The damage and recovery of the PGZ was followed using anti-PCNA antibody, a marker of dividing cells, as previously described by our group [32] (data not shown). At no point of the regenerative process did we detect any significant change in the Sox10^+^ ONH cell population (p>0.05, Fig 3A) or in its distribution in the ONH (Fig 3B–3D).

In contrast, after 7d we found MBP^-^ areas in the ONH. This coincided with the observation that none or very few Zn8^+^ RGC axons were detected at this regenerative stage (Fig 3E). At 15-21d we detected larger MBP^-^ areas, which were mostly located in the ONH (Fig 3F). At the same time, the PGZ regenerated and new Zn8 regenerating RGC axons reached the ONH (Fig 3F). After 30-60d, the ONH recovered the MBP^+^ labeling, leaving the Zn8^+^ growing edge unmyelinated (Fig 3G).

**Fig 4:**
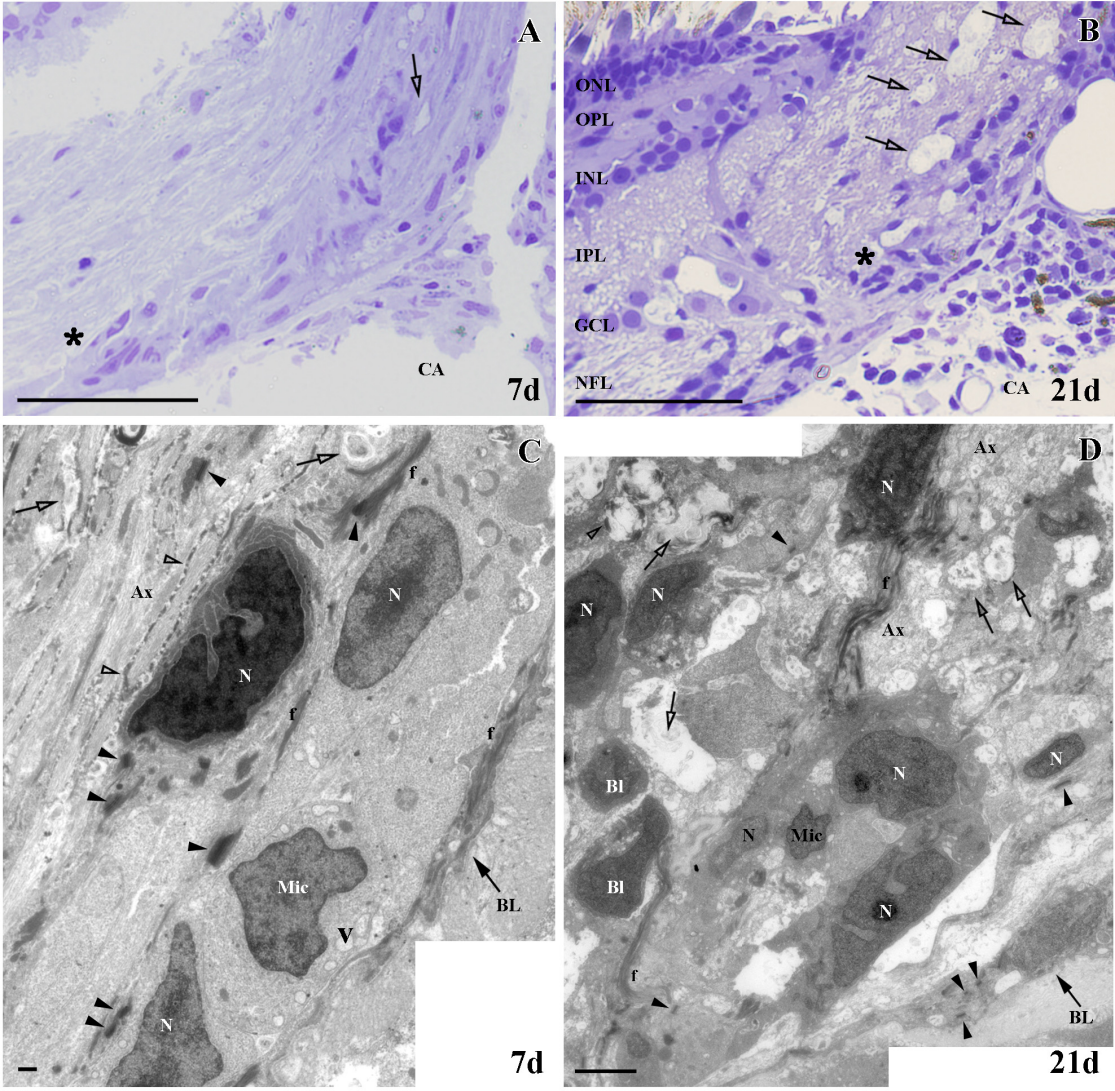
Ultrastructural study of ONH after PGZ cryolesion. **(A-B)** Areas in degeneration (empty arrows) and the areas analyzed in C and D are denoted with an asterisk. **(C)** Small degenerating areas (empty arrows), microglial cells (Mic) and cells in different stages of maturation whose nuclei are indicated with an N. **(D)** Many areas in degeneration (empty arrows), microglial cells (Mic) and blood cells (Bl). Ax: unmyelinated axons, BL: basal lamina, CA: central artery, f: intermediate filaments, GCL: ganglion cell layer, INL: inner nuclear layer, IPL: inner plexiform layer, N: nucleus, NFL: nerve fiber layer, ONL: outer nuclear layer, OPL: outer plexiform layer, V: vesicles, myelin (empty arrowheads), desmosomes (arrowheads). Scale bars: A-B: 50 μm, C: 500 nm; D: 100 nm.

We found similar results in both semithin and ultrathin sections. At 7d, we noted the presence of small degenerative areas in the ONH in semithin sections (Fig 4A), which increased at 21d (Fig 4B). After 7d, in ultrathin sections the tissue surrounding these small degenerating areas displayed a well-organized appearance (Fig 4C). In these regions we also detected some cells featuring the typical characteristics of microglial cells, such as heterochromatic and irregular nuclei and cytoplasmic vesicles. We also found cells showing a different pattern of chromatin packaging and a rich cytoplasm, suggesting oligodendrocytes in different stages of maturation (Fig 4C). In contrast to 7d, after 21d the tissue was much more disorganized with a larger number of degenerating areas (Fig 4D). We found more microglia and blood cells incorporated to the nerve tissue. Similar to 7d, we also observed cells in different stages of maturation (Fig 4D).

### Sox10^+^ ONH oligodendrocytes after ON crushing

In previous work carried out by us we have shown that Pax2^+^ astrocytes in the ONH may have a relevant function in axon guidance when ON crushing was performed [34]. In contrast to PGZ cryolesion, after ON crushing most of the damaged RGCs survive, although they have to regenerate their axons and re-establish their retinotectal connections [40]. In order to investigate the role of Sox10^+^ cells in the ONH in a de- and regenerative environment, we performed ON crushing as a second lesion paradigm.

**Fig 5:**
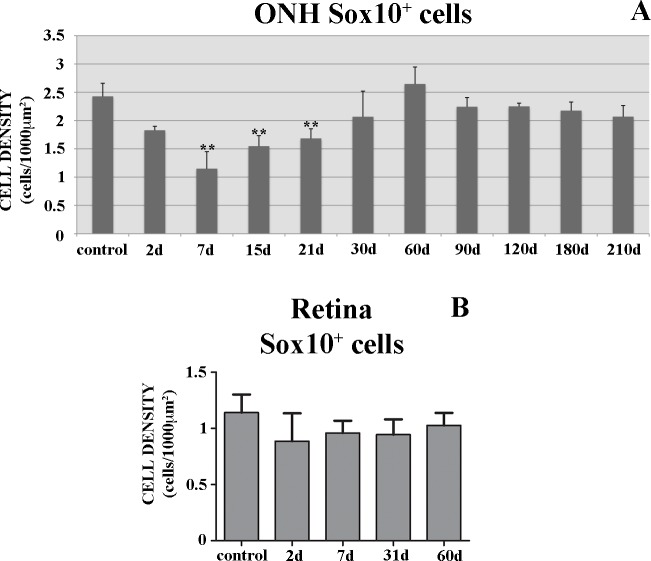
Sox10 time-course analysis after ON crushing. **(A)** ONH Sox10^+^cell counts in sections. **(B)** Retinal Sox10^+^ cells in whole-mount retinas. **p<0.01. Data are shown as means± standard deviations. n = 4 animals per group.

**Fig 6:**
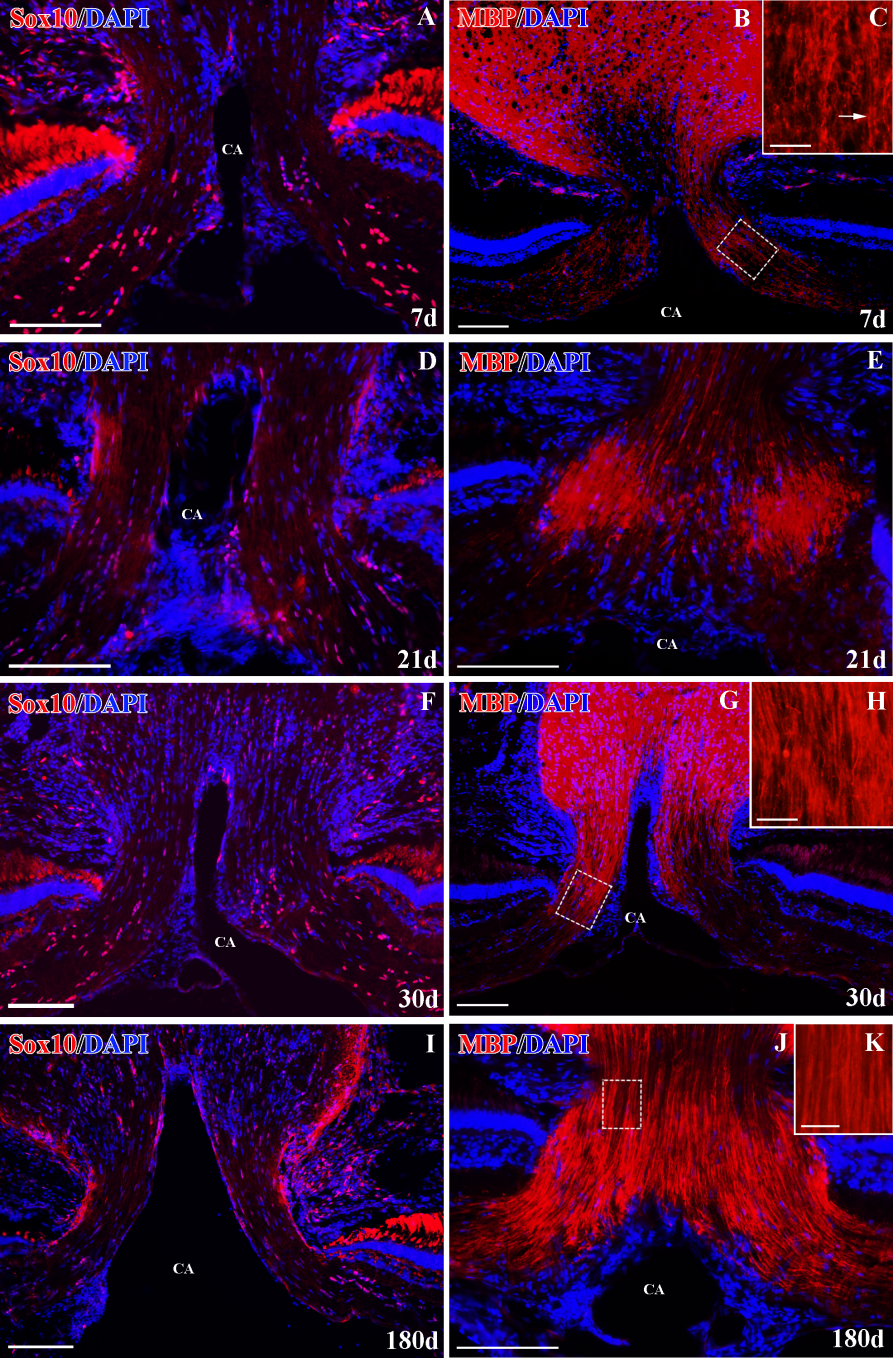
Sox10^+^ cells and MBP axons^+^ in the ONH after ON crushing. **(A-B)** Sox10^+^ cells and MBP^+^ staining at 7d. The square in B is enlarged in C. **(C)** MBP^+^ disorganized axon bundles (arrow). **(D-E)** Sox10^+^ cells and MBP^+^ staining at 21d. **(F-G)** Sox10^+^ cells and MBP^+^ staining at 30d. The square in G is enlarged in H. **(H)** MBP^+^ axon bundles begin to show an organized appearance. **(I-J)** Sox10^+^ cells and MBP^+^ staining at 180d. The square in J is enlarged in K. **(K)** Organized MBP^+^ axons. Cells were counterstained with DAPI (blue). CA: central artery. Scale bars: A-B, D-G, I-J: 100 μm; C, H and K: 20 μm.

We detected a highly significant decrease in the number of Sox10^+^ cells (**p<0.01, Figs 5A and 6A) in the ONH sections after 7d. We found a marked decrease in MBP^+^ immunolabeling at the same degenerative stage (Fig 6B). The remaining weak MBP^+^ labeling in the ONH appeared highly disorganized (Fig 6C). There were no changes in the retinal Sox10^+^ cells (Fig 5B).

After 15-21d, the number of Sox10^+^ cells was still significantly lower than in the control animals (**p<0.01, Figs 5A and 6D) and MBP^+^ labeling was still very weak and disorganized (Fig 6E). From 30d onwards, the number of Sox10^+^ cells was similar to control fish (p>0.05, Figs 5A, 6F and 6I) and we detected more extensive MBP^+^ areas (Fig 6G). Also, the MBP^+^ myelin sheaths began to look organized (Fig 6H). At 180d, we detected fully recovered MBP^+^ labeling, revealing well-organized myelin sheaths (Fig 6J–6K).

**Fig 7:**
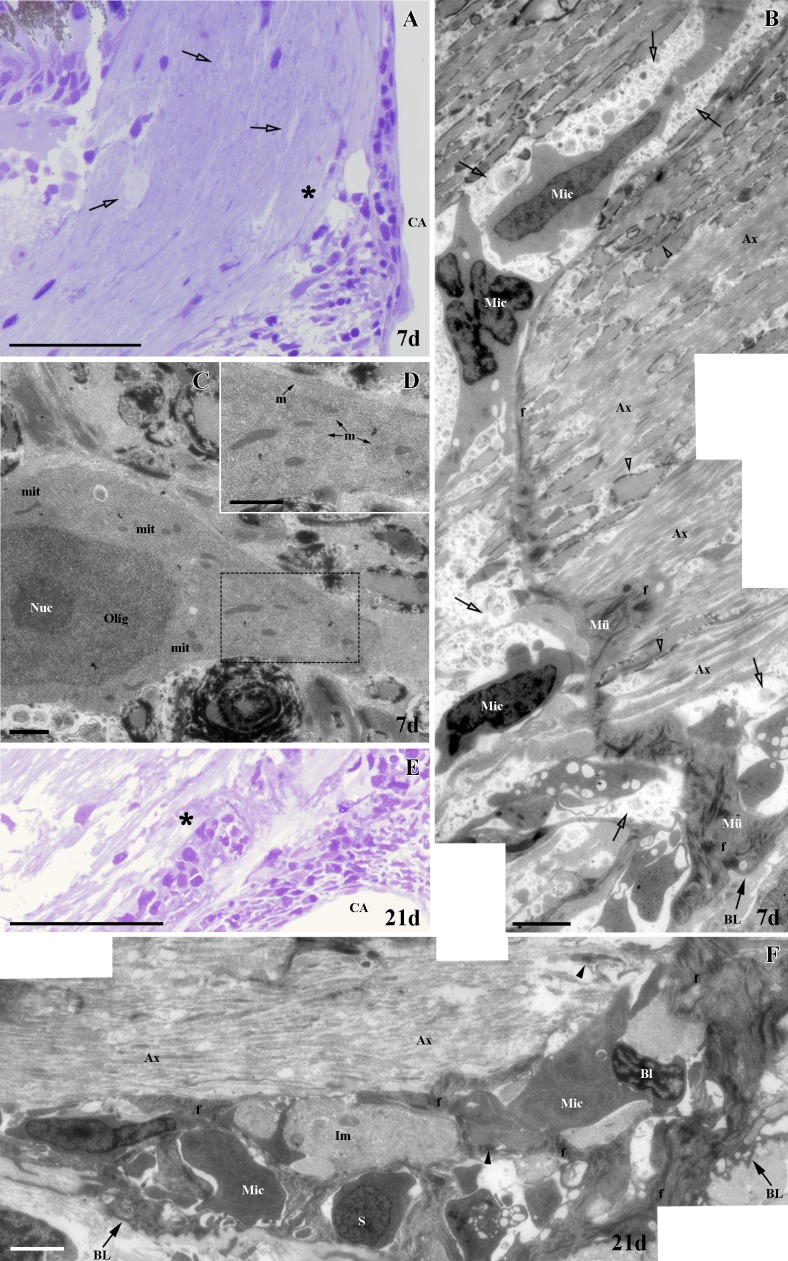
Ultrastructural study of ONH after ON crushing. **(A)** Areas in degeneration (empty arrows) and the area analyzed in B (asterisk). **(B)** The micrograph shows areas in degeneration (empty arrows), a large number of unmyelinated axons (Ax) and few myelinated axons (empty arrowheads). Microglia cells (Mic) associated with Müller end feet (Mü) are detected. **(C)** Oligodendrocyte (Olig). Square enlarged in D. **(D)** Microtubules (m). **(E)** Area analyzed in F (asterisk). **(F)** The micrograph shows a large number of unmyelinated axons (Ax). Numerous microglia cells (Mic), blood cells (Bl) and astrocyte processes with intermediate filaments (f) are seen, as well as the cytoplasm of some immature cells (Im). BL: basal lamina, CA: central artery, f: intermediate filaments, mit: mitochondria, Nuc: nucleolus, desmosomes (arrowheads). Scale bars: A, E: 50 μm; B, F: 2500 nm; C-D: 1000 nm.

In contrast to cryolesion, in semithin and ultrathin sections we found fewer areas undergoing degeneration in the ONH at 7 and 21d (Fig 7A, 7B, 7E and 7F). Interestingly, and consistent with the MBP^+^ labeling described above, at 7d most RGC axons appeared unmyelinated, and the few that did have a thin myelin sheath were mainly located at a considerable distance from the CA (Fig 7B). Similar to the situation observed after cryolesion, we detected high numbers of microglia and blood cells (Fig 7B). Finally, at 7d we found some cells with a euchromatic nucleus and a nucleolus. These cells had a rich cytoplasm with an abundance of microtubules, a typical feature of immature oligodendrocytes (Fig 7C and 7D). We found similar results at 21d. Some immature cells were also detected (Fig 7F).

### Sox10^+^ cell proliferation in the ONH

**Fig 8:**
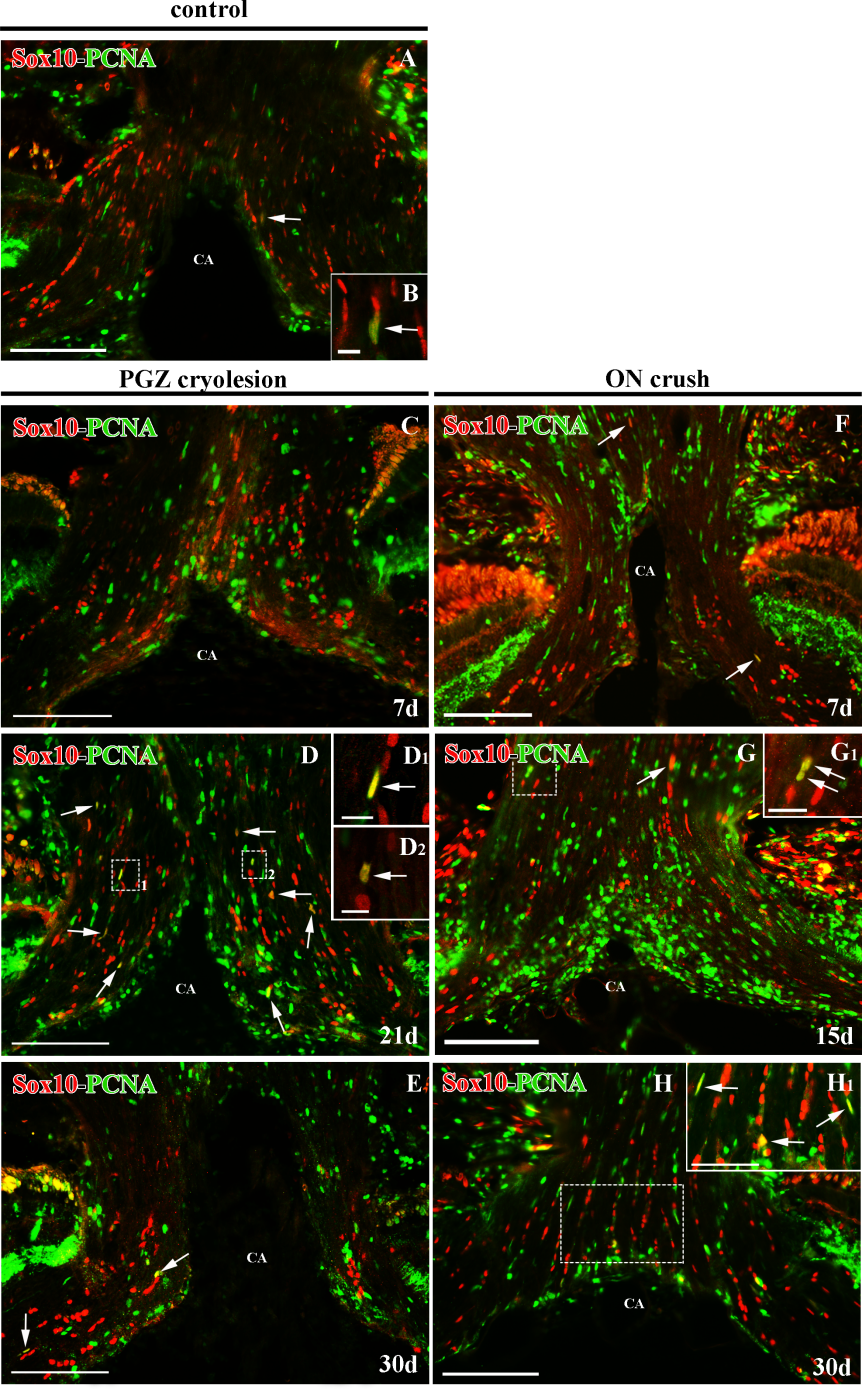
Sox10 and PCNA immunostaining in the ONH of control animals after PGZ cryolesion or ON crushing. Sox10^+^/PCNA^+^ colocalizations are indicated with arrows. **(B)** Shows a detail of Sox10^+^/PCNA^+^ cell present in **(A).** Squares 1 and 2 in **(D)** are enlarged in **(D**_**1**_**)** and **(D**_**2**_**)** respectively, and squares in **(G)** and **(H)** are enlarged in **(G**_**1**_**)** and **(H**_**1**_**)** respectively. All of them show details of Sox10^+^/PCNA^+^ cells. CA: central artery. Scale bars: A, and C-H: 100 μm; H_1_: 50 μm; G_1_: 20 μm; B, D_1_-D_2_: 10 μm.

In control ONH, we found scarce proliferating Sox10^+^/PCNA^+^ cells located among the RGC axons (Fig 8A and 8B). After both types of injury we detected a slight qualitative increase in Sox10^+^/PCNA^+^ cells in the ONH. While in cryolesioned animals we did not note this increase until 21d after injury (Fig 8D, 8D_1_ and 8D_2_), in the ON-crushed animals we detected it earlier, at 7d (Fig 8F). In both cases, we were able to identify these proliferating Sox10^+^ cells throughout the regenerative processes (Fig 8C–8H).

### Localization and distribution of Sox10^+^ cells in the ON

**Fig 9:**
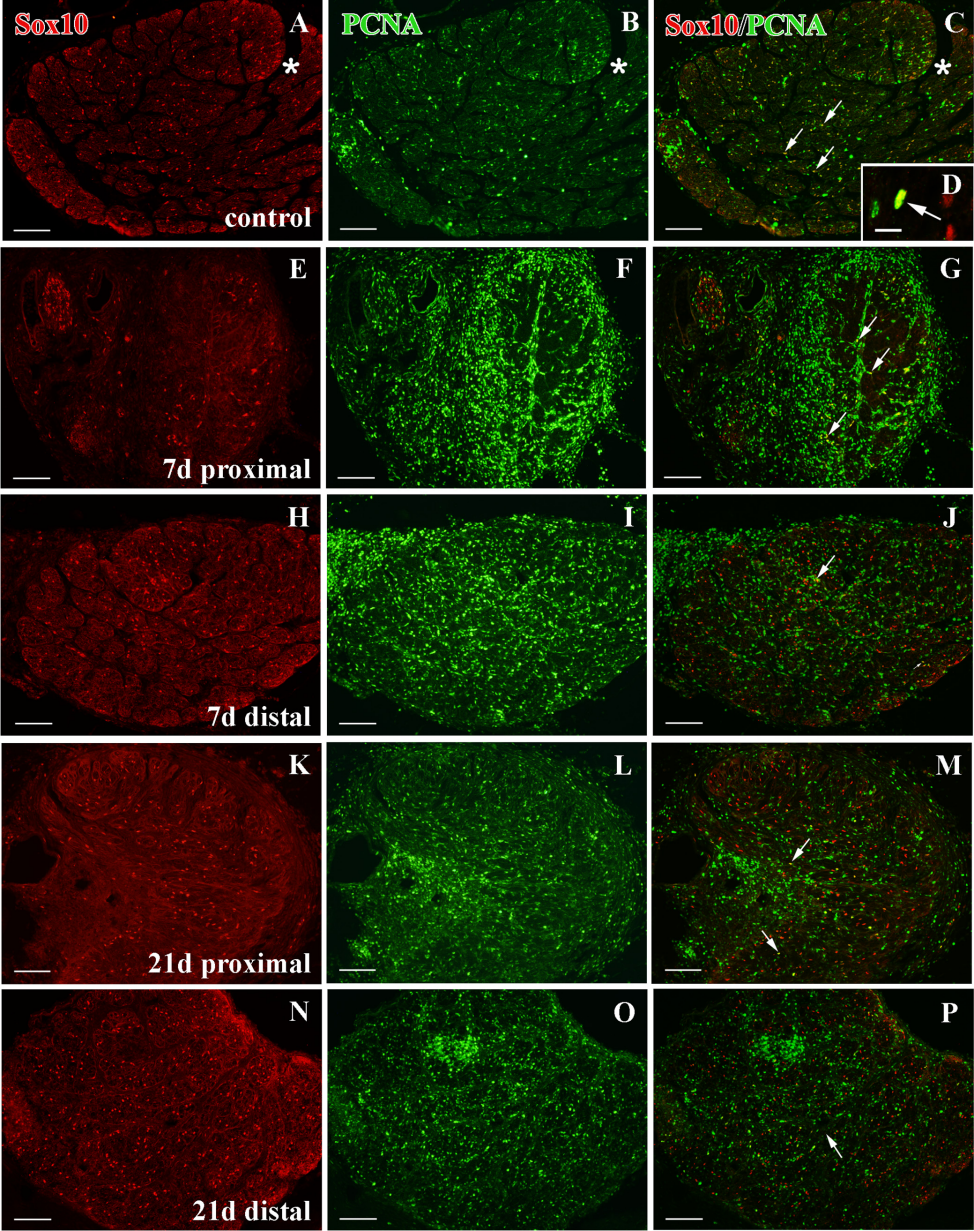
Sox10 and PCNA immunolabeling in the ON after crushing. **(A-C)** Sox10-PCNA staining in control ON cross sections. Sox10^+^/PCNA^+^ cells are in yellow (arrows). The asterisk indicates the growing edge. **(D)** Detail of a Sox10^+^/PCNA^+^ cell (arrow). **(E-G)** Few Sox10^+^ cells are detected in the proximal ON after 7d and very few are also PCNA^+^ (yellow) (arrows). **(H-J)** Sox10-PCNA labeling in the distal ON after 7d. Sox10^+^/PCNA^+^ cells are in yellow (arrows). **(K-M)** Sox10^+^ cells populate the proximal ON after 21d. Some of them are PCNA^+^ (yellow) (arrows). **(N-P)** Sox10-PCNA immunostaining in the distal ON after 21d. Some cells are Sox10^+^/PCNA^+^(yellow) (arrow). Scale bars: A-C, E-P: 100 μm; D: 10 μm.

**Fig 10:**
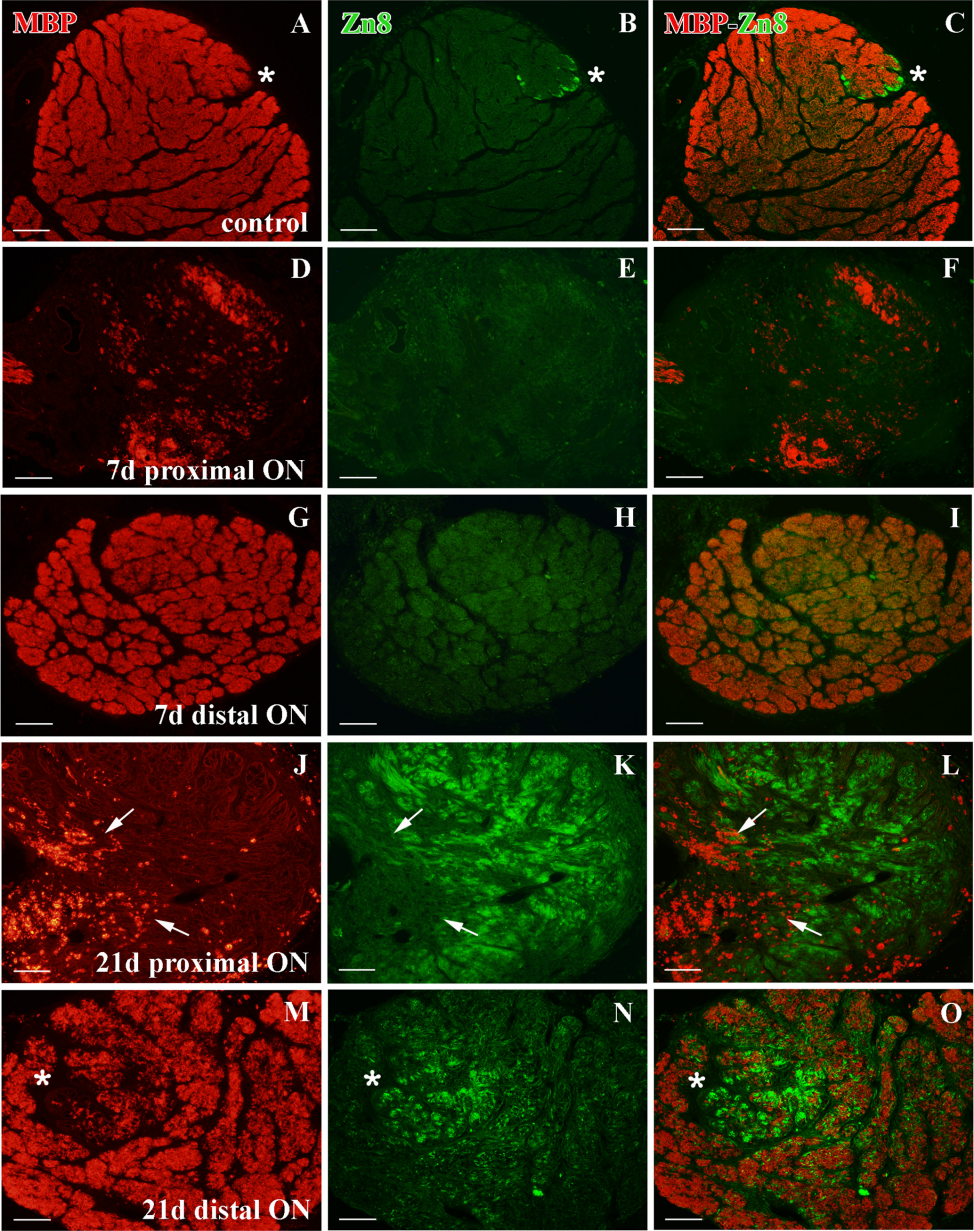
MBP and Zn8 immunolabeling in the ON after crushing. **(A-C)** Zn8^+^ unmyelinated axons (asterisk) versus MBP^+^ myelinated axons in control animals. **(D-F)** No Zn8^+^ axons and a decrease in the MBP^+^ labeling in the proximal ON after 7d. **(G-I)** In the distal ON at 7d after crushing, no Zn8^+^ axons are detected and the MBP labeling seem to be intact. **(J-L)** After 21d the proximal ON receives a large number of Zn8^+^ axons and some degenerating MBP^+^ myelin sheaths are detected (arrows). **(M-O)** A large area of Zn8^+^ axons appears in the distal ON (asterisk) after 21d. These axons are MBP^-^. Scale bars: A-O: 100 μm.

Sox10^+^ cells were distributed throughout the whole ON (Fig 9A). The MBP labeling revealed that the ON was fully myelinated, leaving only the Zn8^+^ growing edge axons unlabeled (Fig 10A–10C). In control ON, we also found scarce Sox10^+^/PCNA^+^ cells (Fig 9A–9D).

### Sox10^+^ cells after ON crushing

In contrast to the cryolesioned animals, where Sox10^+^ cells did not change in the ON during regeneration (data not shown), we found important modifications in ON-crushed animals.

After 7d, we detected scarce Sox10^+^ cells proximal to the crushed area, some of them Sox10^+^/PCNA^+^ (Fig 9E–9G). At the same time-point, most of the region was unmyelinated and we only found some degenerating MBP^+^ areas (Fig 10D). Furthermore, we failed to detect any growing Zn8^+^ axons (Fig 10E and 10F). In contrast, in the distal ON, Sox10^+^ cells were distributed as in the control animals and some of them were also Sox10^+^/PCNA^+^ (Fig 9H–9J). This region was highly myelinated, although some areas appeared with a less intense labeling (Fig 10G). As in the crushed area, we did not find Zn8^+^ axons in this region (Fig 10H and 10I).

After 21d, we detected a higher amount of Sox10^+^ cells proximal to the injured area, some of them colocalizing with PCNA (Fig 9K–9M). Also, only small degenerating MBP^+^ areas appeared in this region (Fig 10J) and many Zn8^+^/MBP^-^ regenerating RGC axons populated the proximal ON (Fig 10J–10L). In the distal ON, we found again Sox10^+^ cells distributed throughout the whole section, together with some Sox10^+^/ PCNA^+^ cells (Fig 9N–9P). A larger unmyelinated area appeared in the distal ON, which was occupied by Zn8^+^/MBP^-^ regenerating axons. After 21d, the remaining distal ON contained MBP^+^ axons (Fig 10M–10O).

From 30d onwards, myelin sheaths began to appear organized and Zn8^+^ axons were restricted to the growth zone. At 180-210d, the ON had completely recovered (data not shown).

## Discussion

Here we analyzed Sox10^+^ cells for the first time in the fish retina and ON, using the goldfish as an animal model. In the development of the CNS and PNS of both mammals and fish Sox10 is present in the oligodendrocyte lineage [1–5], and in mammals it has been demonstrated that Sox10 is expressed in mature CNS oligodendrocytes [2]. In this work we demonstrate that Sox10 is present in retinal and ON oligodendrocytes in adult goldfish. Moreover, we report the behavior of Sox10^+^ oligodendrocytes in the ONH and ON after retinal cryolesion and ON crush, and show that they behave in a similar way to other oligodendrocyte protein markers during ON regeneration [19,41–43].

### Sox10 as an oligodendrocyte marker in the visual system

The transcription factor Sox10 is a highly conserved protein that has been described in zebrafish, mice and humans [1–5]. It participates actively in the specification of the oligodendrocyte lineage and the differentiation and survival of these cells, as it is expressed from OPCs to mature, myelinating oligodendrocytes [1–4]. In the latter ones, it is involved in the expression of some myelin genes, such as MBP; and in PNS myelination [1,2], and it is thought to mediate oligodendrocyte-axon interactions indirectly [4]. Here, we used an antibody designed to specifically recognize zebrafish Sox10 [4,44] in order to locate goldfish oligodendrocytes in the visual system. We also show that Sox10 is located in the nuclei of cells in the retina NFL, in the ONH, and in the ON. This location differs from that observed for astrocytes, characterized by the expression of the Pax2 transcription factor. Pax2^+^ astrocytes are not detected in the goldfish retina, and in the ONH they mainly form the glia limitans [39]. By contrast, Sox10^+^ cells were distributed from areas close to the PGZ to the whole length of the ON. Furthermore, Sox10^+^ cells were mainly organized in groups and rows. This particular organization has been described previously in tench oligodendrocytes using electron microscopy techniques [17,18]. Upon examining the ultrastructure of these rows in goldfish, they showed the same characteristics. Thus, we can affirm that goldfish Sox10^+^ cells are oligodendrocytes.

### Oligodendrocyte location in the retina

In this study we detected the presence of both Sox10^+^ oligodendrocytes and MBP^+^ axons in the NFL of the goldfish retina. Sox10^+^ cells were organized in groups and in rows, and they were distributed throughout the whole ONH and retina. We also found that retinal MBP^+^ axon bundles had a loose appearance compared with the compact axon bundles detected in the ONH and ON. This is consistent with previous electron microscopy analyses carried out in other fish species [15,17,18,43]. Besides the expression of Sox10 and MBP described here, retinal oligodendrocytes express other proteins and genes related to the oligodendrocyte lineage in zebrafish. Examples of this are Contactin1a, only expressed in differentiating oligodendrocytes [43]; A20 antigen, which recognizes a meshwork surrounding myelinated axons [45]; Claudin k, located in the mesaxon, and the transcription factor Olig2 [19]. However, these oligodendrocytes fail to express P0, a marker of compact myelin [42].

The role of these retinal oligodendrocytes is still unclear, although different theories have been proposed. In birds, which also have oligodendrocytes in both the NFL and the GCL, oligodendrocytes are believed to participate not only in the myelination but also in the nutrition and protection of ganglion cells [22,24]. In lizards, these retinal oligodendrocytes seem to be involved in re-myelination processes after surgical lesion, and it has been proposed that the loose myelin sheaths help to maintain retinal transparency [21]. In fish, it has been suggested that environmental factors would prevent retinal oligodendrocytes from forming compact myelin [19]. All the data taken together indicate that goldfish retinal oligodendrocytes seem to maintain certain aspects of immature oligodendrocytes that allow the continuous growth of the visual system and its ability to regenerate.

### Sox10^+^ oligodendrocytes in regeneration

We have previously shown that ONH Pax2 astrocytes play an important role not only in retinal regeneration [33] but also after ON crushing [34]. Since the fish retina contains mature myelinated axons and immature unmyelinated axons [15], we aimed to analyze the behavior of ONH oligodendrocytes when deprived of new unmyelinated axons (PGZ cryolesion) and when large numbers of myelinated axons and their myelin sheaths are destroyed (ON crushing). Many authors have analyzed the role of oligodendrocytes and myelin-related proteins in the de- and regeneration and remyelination of RGC axons in the ON after an injury [19,35,41–43,46–49]; however, little attention has been paid to their behavior in the ONH.

Here we show that after PGZ cryolesion no changes occur in the number and organization of Sox10^+^ oligodendrocytes in the ONH, although we did detect degenerating myelin areas during the first days of regeneration. This was probably due to cell death phenomena, triggered by the elimination of the somata of some RGC in the PGZ. In contrast, after ON crushing, the number of ONH Sox10^+^ oligodendrocytes was markedly reduced during the first days of recovery, although their population did not change in the retina. Also, MBP^+^ labeling decreased and, instead, a large number of regenerating Zn8^+^ axons was observed (data not shown) [40]. These data are especially interesting, since numerous studies have described a fast regenerative response of injured axons in the crushed area, which is distant from the ONH [50–56]. A retrograde degenerating process may first exist prior to regeneration, due to the strong demyelination undergone by the ONH during the first week after crushing. Another more plausible explanation is that the oligodendrocytes could lose contact with the RGC axons as a consequence of ON crushing. In support of this, it has been proposed that the loss of oligodendrocyte-axon contact leads to oligodendrocyte death [4,57,58]. However, whether the oligodendrocytes die, return to an OPC state or dedifferentiate into other type of progenitor, stopping the expression of Sox10, remains to be analyzed.

The results of our analyses of Sox10^+^ oligodendrocytes and MBP in both the crushed area and the distal ON are similar to those of other studies performed on oligodendrocyte-related proteins [19,41–43]. After 7d, we detected few Sox10^+^ oligodendrocytes and few myelin MBP sheaths, which increased after 15-21d. The new myelinating cells that invade the regenerating nerve have been described as Schwann cells [49]. Both oligodendrocytes and Schwann cells express Sox10 [1,2,4], although recent studies support the notion that the cells responsible for myelinating the regenerating axons are oligodendrocytes [19,59].

In both the ONH and ON we found a slight increase in dividing Sox10^+^ /PCNA^+^ cells, and our electron microscopy analyses revealed oligodendrocytes in different stages of maturation. It has previously been demonstrated that oligodendrocytes can be newly generated after ON crushing [19] or from oligodendrocytes that de- and re-differentiate during regeneration [41].

The role of Sox10 in ON regeneration has not yet been analyzed. In other parts of the nervous system, Sox10 has been shown to be involved in the synthesis of myelin proteins, such as MBP [1,2]. Integrins are also candidate targets for Sox10 regulation because they mediate axon-dependent oligodendrocyte survival [4]. Thus, it seems likely that Sox10 plays an active role in fish axon regeneration and remyelination. Further experiments need to be carried out, and as we have shown here the fish visual system, including the ONH, provides a versatile model for the study of oligodendrocytes.

## Conclusions

We have shown that the adult goldfish retina contains Sox10^+^ oligodendrocytes and MBP^+^ loose myelin axons. We also detected mature Sox10^+^ oligodendrocytes in the ONH and ON. We tested their response after two different lesion paradigms: PGZ cryolesion, which destroys the input of unmyelinated axons to the visual system, and ON crushing, which destroys stable, myelinated axons. Their different behavior reveals their extreme plasticity and suggests that the ONH would be an excellent area for further investigation of these cells.

## Supporting Information

S1 Fig Myelinated axons in the retina. MBP^+^ axons (arrows) show a loose appearance in the retina when compared to ONH. Zn8^+^ growing axons (arrow heads) do not overlap with MBP^+^ axons.
(TIF)
