# Supplementary figures and images for: Sox10 Expression in Goldfish Retina and Optic Nerve Head in Controls and after the Application of Two Different Lesion Paradigms
Source: PLoS One. 2016 May 5;11(5):e0154703. doi: 10.1371/journal.pone.0154703 (PMC4858161; doi:10.1371/journal.pone.0154703)

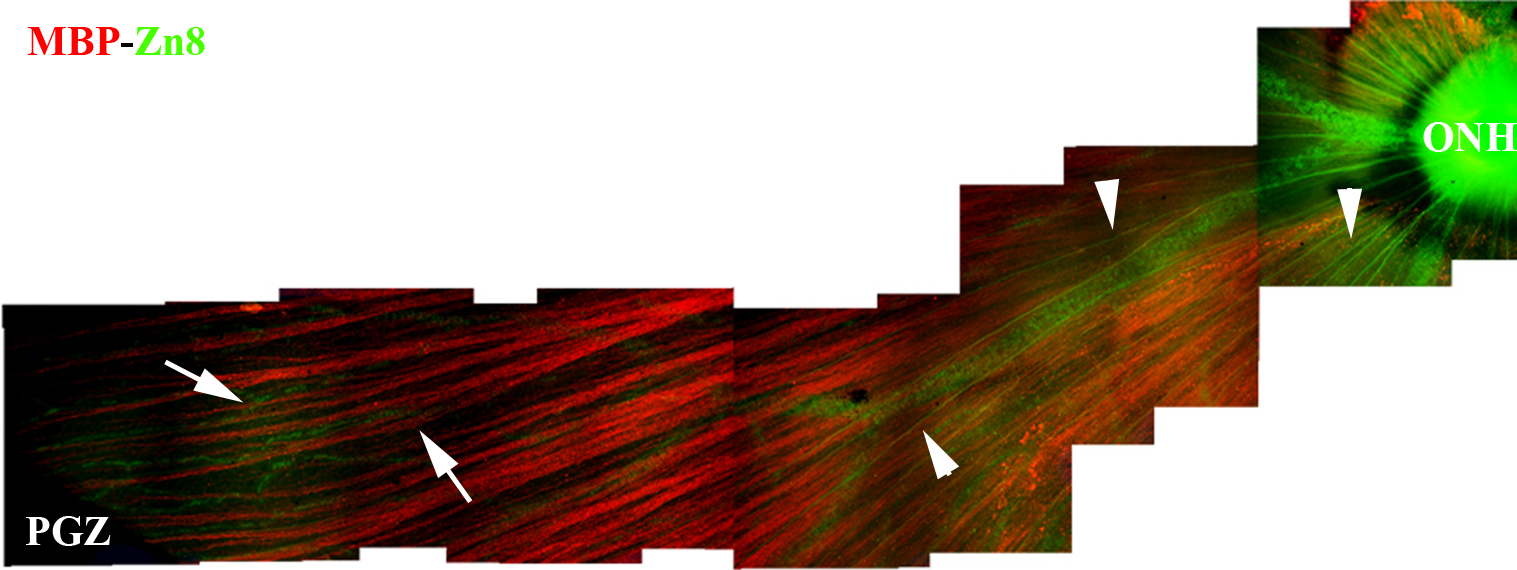

Supplement: S1 Fig — MBP+ axons (arrows) show a loose appearance in the retina when compared to ONH. Zn8+ growing axons (arrow heads) do not overlap with MBP+ axons. (TIF) [file pone.0154703.s001.tif]
